# Draft Genome Sequences of Five Potentially Probiotic Enterococcus faecium Strains Isolated from an Artisanal Tunisian Meat (Dried Ossban)

**DOI:** 10.1128/MRA.01348-19

**Published:** 2020-01-16

**Authors:** Mohamed Zommiti, Mounir Ferchichi, Khaled Sebei, Marc G. J. Feuilloley, Nathalie Connil, Amine M. Boukerb

**Affiliations:** a Unité de Protéomique Fonctionnelle et Potentiel Nutraceutique de la Biodiversité de Tunisie, Institut Supérieur des Sciences Biologiques Appliquées de Tunis, Université de Tunis El-Manar, Tunis, Tunisia; b Laboratoire de Microbiologie Signaux et Microenvironnement (LMSM) EA 4312, Université de Rouen Normandie, Normandie Université, Évreux, France; Loyola University Chicago

## Abstract

Here, we report the first draft genome sequences of five bacteriocinogenic and potentially probiotic Enterococcus faecium strains (MZF1 to MZF5), which were isolated from homemade Tunisian meat (dried ossban). The estimated median genome sizes were about 2,582,641 ± 109,039 bp, with a median G+C content of 40% ± 0.4%.

## ANNOUNCEMENT

*Enterococcus* spp. are Gram-positive lactic acid bacteria that are ubiquitous in nature (soil, water, and plants) but are mostly considered to be of fecal origin, as they are common members of the intestinal microbiota of humans, mammals, reptiles, birds, and insects (1). They are also found in foods (2), especially in a wide range of traditional fermented products (3).

Enterococcus faecium is one of the main species of this genus (4). Its microbial antagonism is related to the production of proteinaceous substances known as enterocins, which are highly active against closely related species and other Gram-positive bacteria (e.g., Listeria monocytogenes) (5). Recently, we isolated five potentially bacteriocinogenic strains of E. faecium from a homemade fermented Tunisian meat called dried ossban (5, 6). Meat samples were collected aseptically from various governorates, covering almost all Tunisian territory. For each sample, 10 g was added to 90 ml of sterile peptone saline water and homogenized through vortexing at maximum speed for 10 min. Appropriate decimal dilutions were plated onto de Man-Rogosa-Sharpe (MRS) agar and incubated at 37°C for 24 to 48 h. A selection of Gram-positive and catalase-negative colonies were assessed by analysis of their total proteome using an Autoflex III matrix-assisted laser desorption ionization–time of flight mass spectrometer (MALDI-TOF MS; Bruker, Marcy-l’Etoile, France) coupled to the MALDI-Biotyper 3.1 algorithmic system for species validation. The five E. faecium strains from dried ossban were identified with this technique, as was another bacteriocinogenic probiotic strain, Pediococcus pentosaceus MZF16 (6, 7), confirming the originality of this North African food for its high potential source of probiotic bacterial strains.

Genomic DNAs were extracted from overnight cultures in MRS broth using a GeneJet genomic DNA purification kit (Thermo Scientific, France), as recommended by the manufacturer, and assessed using the double-stranded DNA (dsDNA) high-sensitivity kit on a Qubit fluorometer (Thermo Fisher Scientific, USA) and 1% agarose gel electrophoresis. Sequencing libraries were prepared using the Nextera XT DNA sample preparation kit (Illumina, USA). Genomic sequencing was performed by the Laboratory of Microbiology Signals and Microenvironment (LMSM) genomics platform (Rouen Normandy University, Evreux, France) using a MiSeq instrument (Illumina) with a 2 × 250-bp paired-end read protocol.

Default parameters were used for all software unless otherwise noted. TrimGalore v.0.6.2 (8) was used for read trimming, and read quality was checked with MultiQC v.1.6 (9). *De novo* assembly was performed with Unicycler v.0.4.7 (10), and assembly metrics were calculated using QUAST v.5.0.0 (11). Structural gene prediction and functional annotation were carried out using the Prokka pipeline v.1.14.0 (12). Antimicrobial mechanisms (e.g., enterocin production) were explored with BAGEL v.4 (13) and antiSMASH v.5 (14). Multilocus sequence typing (MLST), the resistome, and the virulome were assessed using Nullarbor v.2.0.20181010 (15) with sequence identity and coverage thresholds fixed at 70% and 90%, respectively.

The average (± standard deviation) total size of the draft genomes was 2,582,641 ± 109,039 bp arranged into 90 ± 30 contigs (Table 1). The genome sequence data were at a median 189.3 ± 22.9× coverage with a median *N*_50_ value of 90,377 ± 38,346 bp and mean G+C content of 40% ± 0.4%. These genomes contain, on average, 2,444 ± 116 coding DNA sequences (CDSs), 3 rRNAs, 52 ± 2 tRNAs, and 1 transfer-messenger RNA (tmRNA). BAGEL4 and antiSMASH5 predicted six bacteriocin biosynthetic gene clusters associated with antimicrobial activity (enterocins *entA*, *entB*, *entP*, *entX,* and *uviB* and enterolysin A) along with regulatory genes, immunity proteins, and transporters that were differentially detected in the five genomes (Table 1). MLST identified strain MZF3 with new alleles for *pstS* and *adk*, while the other strains belonged to sequence type 22 (ST22). No virulence factors (e.g., *ace*, *acm*, *asc*, *ayl*, *ecbA*, *efaA*, *esp*, *gelE*, *scm*, and *sprE*) that correlate with human pathogenicity were identified. We found two intrinsic antibiotic resistance genes, namely, *aac6′* (aminoglycosides) and *msrC* (macrolides and streptogramin B antibiotics), and one acquired resistance determinant, *eatA* (cross-resistance to lincosamides, streptogramins A, and pleuromutilins). Altogether, the genetic features of these five strains are of interest, serving as candidates for investigation into bacterial antagonism associated with the production of multiple bacteriocins.

**TABLE 1:** Overview of the draft genome assemblies from the five E. faecium MZF strains^*a*^

Parameter	Data for strain:				
	MZF1	MZF2	MZF3	MZF4	MZF5
GenBank accession no.	VOLZ00000000	VOLY00000000	VOLX00000000	VOLW00000000	VOLV00000000
SRA accession no.	SRR9859857	SRR9859856	SRR9859859	SRR9859858	SRR9859855
Total no. of reads	2,387,940	2,646,590	2,674,554	2,079,382	2,399,374
Mean coverage (×)	157.4	212	199.2	165.3	189.3
G+C content (%)	39.4	40.4	40.1	40.3	40.1
*N*_50_ (bp)	128,723	96,021	52,031	84,233	68,415
No. of contigs	74	60	119	94	106
Genome size (bp)	2,691,680	2,532,545	2,473,602	2,651,081	2,639,444
No. of CDSs	2,559	2,375	2,328	2,519	2,483
No. of tRNAs	54	50	51	52	53
No. of rRNAs	3	3	3	3	3
Enterocin(s)	*entA*, *entB*, *entP*, *uviB*, enterolysin A	*entA*, *entX*	*entA*	*entA*, *entB*, *entP*, *uviB*, enterolysin A	*entA*, *entX, uviB*

a All isolated from artisanal Tunisian meat (dried ossban).

### Data availability.

The draft sequences of the genomes have been deposited at DDBJ/ENA/GenBank under the accession numbers cited in Table 1.
